# Supplementary material for: Varied Bulk Powder Properties of Micro-Sized API within Size Specifications as a Result of Particle Engineering Methods
Source: Pharmaceutics. 2022 Sep 8;14(9):1901. doi: 10.3390/pharmaceutics14091901 (PMC9500803; doi:10.3390/pharmaceutics14091901)
Supplement: Supplementary file 1 [file pharmaceutics-14-01901-s001.zip › pharmaceutics-1860634-supplementary.pdf]

Supporting information

Several XRD peaks of sample B1 tablet disappeared in the range of 5 to 15 degrees (Figure S1), suggesting the B1 crystals may have preferred orientation upon compaction. However, all the peaks of sample A3 in this range are consistent with calculated pattern (Figure S1).

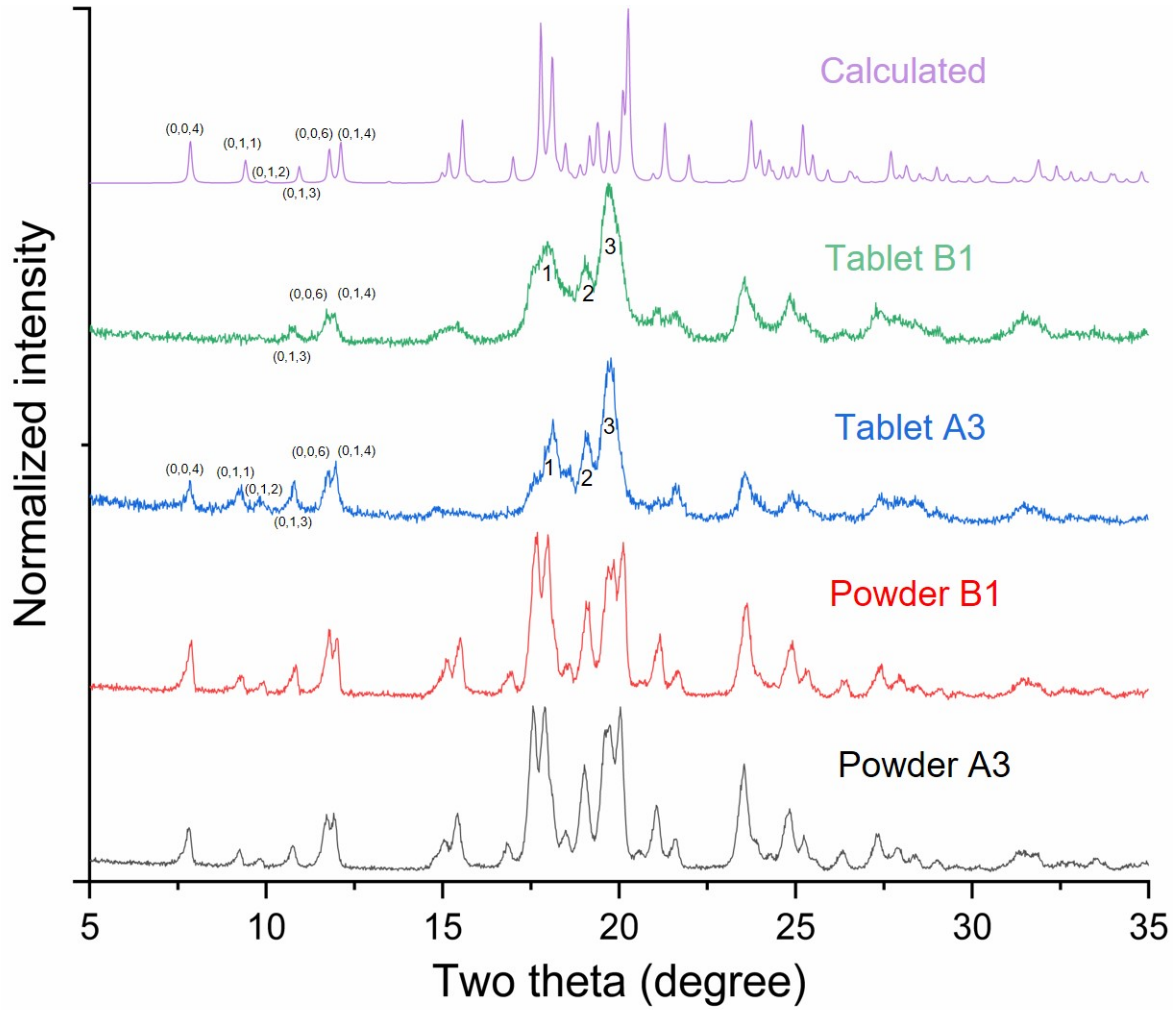

Figure S1. XRD overlap of samples A3 and B1. (Enlargement of Figure 4a)

Table S1. Full width at half maximum (FWHM) analysis of tablets XRD of samples A3 and B1.

	**FWHM (2 theta)**		
**Peak**	**1**	**2**	**3**
Tablet A3	0.68	0.35	0.37
Tablet B1	0.97	0.42	0.89

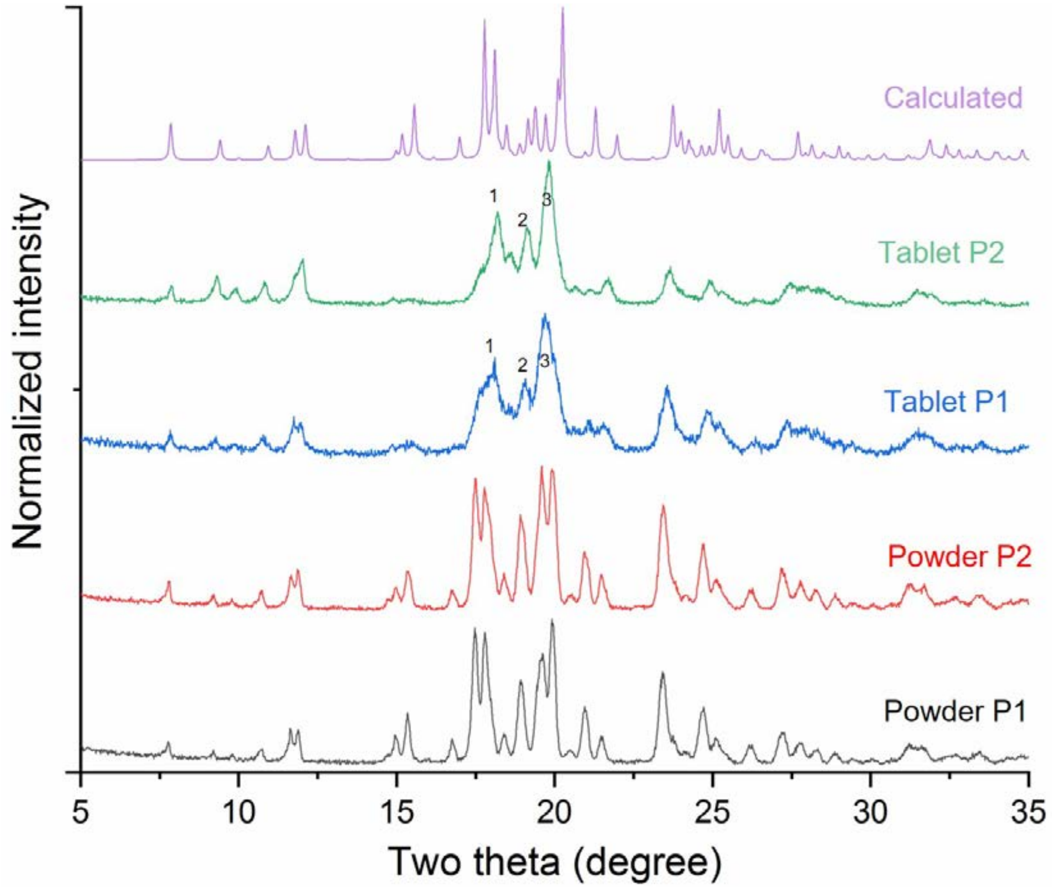

Figure S2. XRD overlap of samples P1 and P2. (Enlargement of Figure 7a)

Table S2. Full width at half maximum (FWHM) analysis of tablets XRD of samples P1 and P2.

Peak	FWHM (2 theta)		
	**1**	**2**	**3**
Tablet P1	0.91	0.39	0.72
Tablet P2	0.50	0.37	0.63

Several XRD peaks of A(M) powder disappeared below 16 degrees and the intensities of existed peaks of powder A(M) are much less than A3 and A(S) powders (Figure S3).

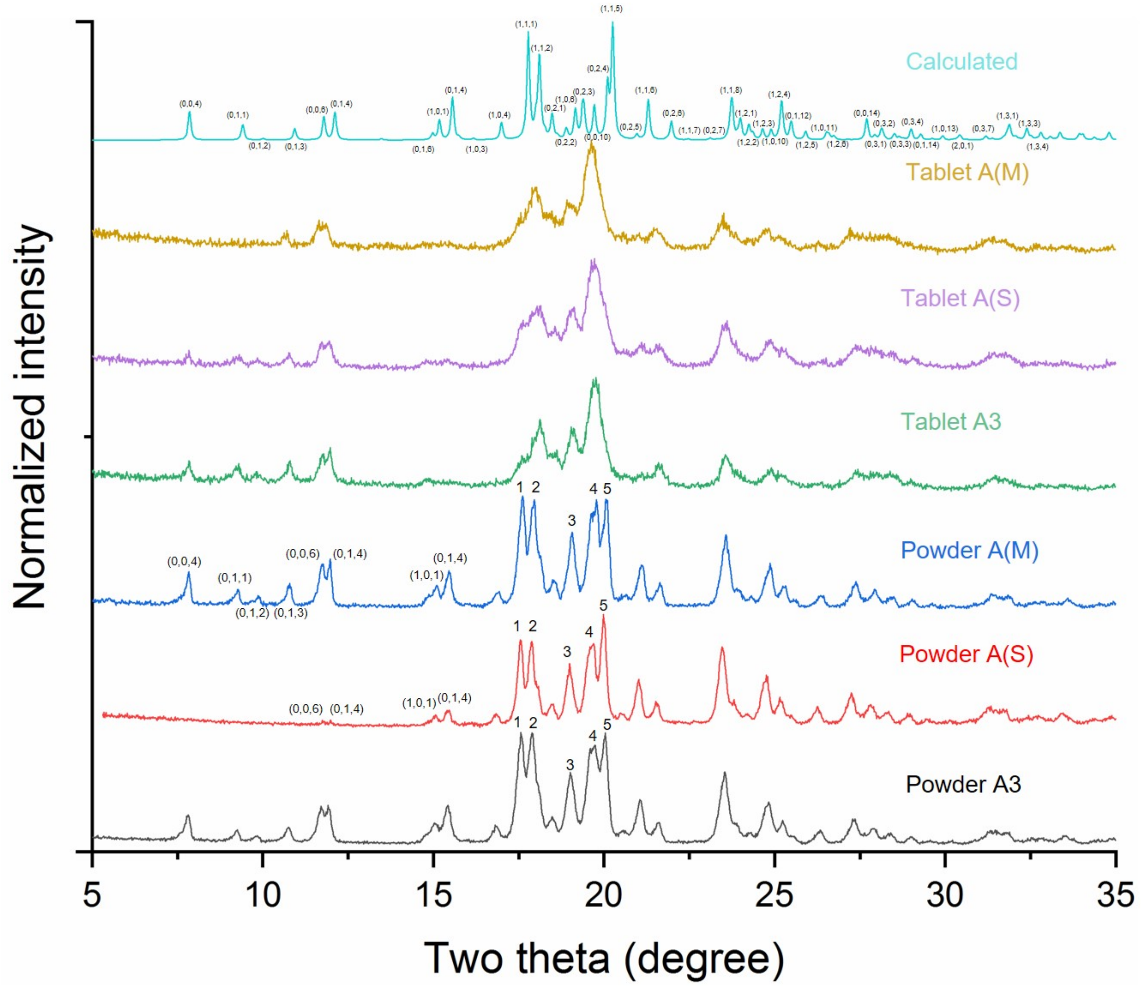

Figure S3. XRD overlap of samples A3, A(S) and A(M). (Enlargement of Figure 9a).

Table S3. Full width at half maximum (FWHM) analysis of tablets XRD of samples A3, A(S), and A(M).

Peak	FWHM (2 theta)				
	**1**	**2**	**3**	**4**	**5**
Powder A(3)	0.23	0.37	0.28	0.41	0.20
Powder A(S)	0.19	0.30	0.25	0.35	0.19
Powder A(M)	0.20	0.32	0.24	0.39	0.17
